# Sensory Neurotization of the Ulnar Nerve, Surgical Techniques and Functional Outcomes: A Review

**DOI:** 10.3390/jcm11071903

**Published:** 2022-03-29

**Authors:** Mỹ-Vân Nguyễn, Jérôme Pierrart, Vincent Crenn

**Affiliations:** 1 Orthopedic and Traumatology Unit, Nantes University Hospital, 1 Place Alexis Ricordeau, 44000 Nantes, France; myvan_clara@yahoo.fr; 2 Institut de la Main Nantes-Atlantique, Santé Atlantique, Avenue Claude Bernard, 44800 Saint-Herblain, France; 3 Cabinet Archimed, SOS Mains Côte d’Opale, Clinique des 2 Caps, 80 Avenue des Longues Pièces, 62231 Coquelles, France; pierrartjerome@gmail.com; 4 CRCI2NA (Centre de Recherche en Cancérologie et Immunologie Nantes-Angers), INSERM UMR 1307, CNRS UMR 6075—Team 9 CHILD (CHromatin and Transcriptional Deregulation in Pediatric Bone Sarcoma), Nantes Université, 1 rue Gaston Veil, 44035 Nantes, France

**Keywords:** ulnar nerve, sensory neurotization, surgical technique, nerve transfer, BMRC, functional outcomes

## Abstract

When ulnar nerve lesions happen above the wrist level, sensation recovery after acute repair or nerve grafting is often challenging. Distal sensory nerve transfers may be an option for overcoming these sequelae. However, little data has been published on this topic. This study aims to review the surgical procedures currently proposed, along with their functional results. Six donor nerves have been described at the wrist level: the palmar branch of the median nerve, the cutaneous branch of the median nerve to the palm with or without fascicles of the ulnar digital nerve of the index finger, the posterior interosseous nerve, the third palmar digital nerve, the radial branch of the superficial radial nerve, the median nerve, and the fascicule for the third web space. Three donor nerves have been reported at the hand level: the ulnar digital nerves of the index, and the radial or ulnar digital nerves of the long finger. Three target sites were used: the superficial branch of the ulnar nerve, the dorsal branch of the ulnar nerve, and the ulnar digital branch of the fifth digit. All the technical points have been illustrated with anatomical dissection pictures. After assessing sensory recovery using the British Medical Research Council scale, a majority of excellent recoveries scaled S3+ or S4 have been reported in the targeted territory for each technique.

## 1. Introduction

Ulnar nerve injuries are the most common peripheral nerve injuries of the upper limb. Collected data between 1993 and 2006 across the United States revealed up to 55,739 ulnar nerve injuries, ranking it above brachial plexus, median, and radial nerve lesions. The health care costs associated with these lesions range from $10,563 to $42,000 per individual [1]. The vast majority of these patients are aged 18 to 44 years [1], underlining the economic and social impact in case of poor recuperation.

Specifically, functional outcomes of the acute repair rely not only on motor but also on sensory recovery. In addition, both recoveries are interlinked as tactile gnosis cannot be improved without voluntary motor activity [2].

However, impaired sensibility is often reported after distal ulnar nerve lesions. Despite a microsurgical fascicular epineural 10-0 suture, within 24 h of ulnar nerve neurotmesis, long-term results showed that 55% of patients had not regained any protective sensation [3]. Following an interfascicular sural nerve graft, only 28% of the patients were able to report either none or a two-point discrimination test between 10 to 13 mm after two years of minimal follow-up [4]. While the most common cause of impaired ulnar sensibility remains acute traumatic separation of the nerve, patients with low brachial plexus injuries involving the T1 and possibly C8 and C7 roots [5] or leprosy sequelae could also benefit from ulnar sensory reconstruction. Restoring at least a protective sensation to the ulnar border of the hand allows it to play its role of stabilization and support for the hand while the radial fingers carry out manipulation. Loss of sensation on the ulnar side of the hand leads to injuries such as repeated self-mutilation [6] and functional impairment.

There are many ways to assess sensory recovery after peripheral nerve surgery. The British Medical Research Council (BMRC) Scale, modified by Mackinnon and Dellon, is commonly used [7]. It evaluates the recovery of deep and superficial cutaneous pain, along with tactile gnosis, using static (S2PD) and moving (M2PD) two-point discrimination (2PD) tests. Two-point discrimination is an assessment tool for tactile gnosis. First described by Moberg in 1958, S2PD reports the smallest distance at which the patient was able to discriminate 7 out of 10 random applications of one or two points. Contrary to S2PD, M2PD implies moving the calipers over the skin’s surface [8,9]. Whereas S2PD is thought to measure the innervation density of the slowly adapting receptors, M2PD relies on the quickly adapting receptor systems [10] and its threshold has been demonstrated to be lower than those of the S2PD [11]. The BMRC scale for sensation is categorized from S0 to S4: S0 refers to the absence of sensation, while S4 is complete recovery with S2PD between 2–6 mm and M2PD between 2–3 mm. The detailed scale is developed in Table 1 (adapted from Wang et al. 2013 [10]).

Over the past 20 years, several motor nerve transfer procedures have been described to restore ulnar nerve palsy. The intrinsic muscles regained sufficient power to act against gravity or even better in 70% of cases after transferring the pronator quadratus branch from the anterior interosseous nerve to reinnervate the deep motor branch of the ulnar nerve for intrinsic muscles [12]. Nevertheless, given the priority for motor recovery, only a few studies have focused on surgical strategies to deal with a loss of sensation, and published data on ulnar sensory neurotization remain rare.

This article first intends to review the current techniques described for sensory neurotization of the ulnar nerve, following lesions above the wrist level. Secondly, it aims to summarize their functional outcomes according to the BMRC Scale, modified by Mackinnon and Dellon, which is our primary outcome parameter [10]. The secondary outcomes parameters focus on donor site morbidity including paresthesia, anesthesia, discomfort, pain, loss of protective sensory function, adverse sensation affecting daily activities, or any subjective abnormal sensation reported.

## 2. Materials and Methods

We used the MeSH terms: “ulnar nerve”, and the keywords “sensory” and “nerve transfer”. The MEDLINE database was used.

The inclusion criteria were: all original articles on sensory neurotization of the ulnar nerve in the wrist or the hand following a proximal ulnar nerve lesion above the wrist level, which cannot be repaired in situ: large nerve defect after tumoral nerve resections, extensive crush or burn injury with severe scarring of the nerve, low C7-T1 brachial plexus injury with chronic impairment in ulnar nerve sensory territory, low ulnar nerve palsy, chronic (i.e., more than six months since the initial repair) sequelae of traumatic nerve separation, and leprosy sequelae in the ulnar nerve territory.

The exclusion criteria were: finger amputations, injuries that were expected to recover spontaneously (i.e., axonotmesis), established chronic illnesses such as Charcot-Marie-Tooth syndrome, diabetes, connective tissue diseases, dementia, or cognitive disorders, as these could impair the sensory rehabilitation following the surgery. We also excluded the articles with cadaveric studies only, functional outcomes that were not measured using the BMRC Scale, surgical procedures with nerve graft interposition, neurovascular skin island flaps, nerve transfers above the elbow level, cubital tunnel syndrome, and studies assessing only the motor function of the ulnar nerve. We completed our research by adding the articles cited after a full review of the selected papers. The last electronic research was carried out in December 2021 and was not limited by the year of publication. Only studies available in English or French were considered for review. Data were extracted independently. The publication types were as follows: case reports, evaluation studies, and comparative studies.

Dissections were performed in the anatomy department of our surgical school (Ecole de Chirurgie du Fer à Moulin, Paris) on frozen adult human cadavers with no known disease or trauma. Six upper arms were carefully dissected (four males, two females) aged from 67 to 83 years old.

## 3. Results

**Figure 1:**
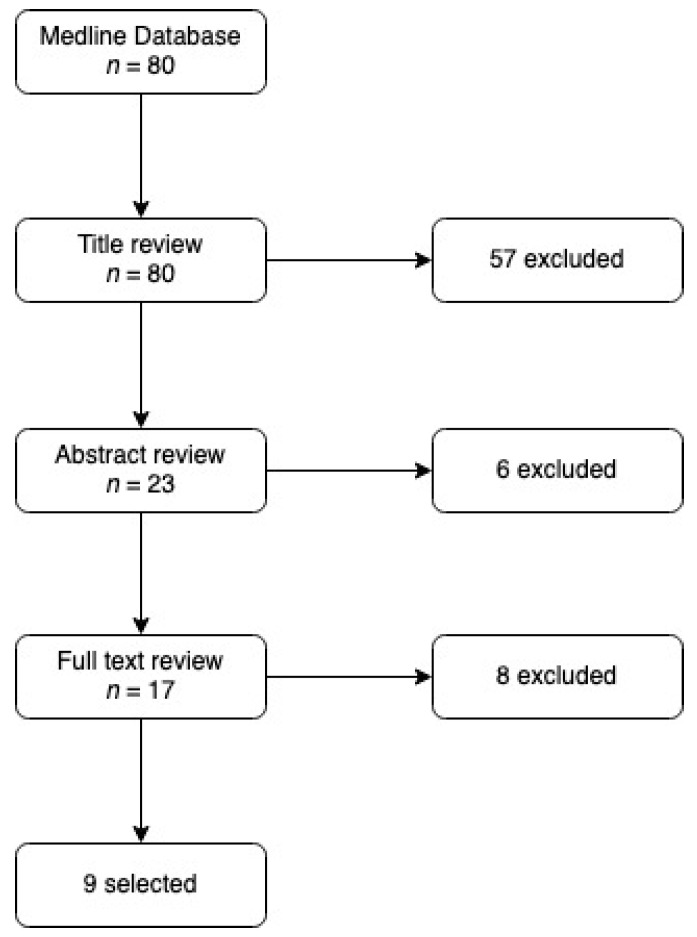
Flow diagram of the review process.

Our search strategy produced 80 studies. After exclusion of duplicates and implementation of inclusion and exclusion criteria, we took on nine articles. A flow diagram of this process is shown in Figure 1.

### 3.1. Population

The sensory neurotization of the ulnar nerve remains a seldom performed surgical procedure: small cases series have been reported ranging from case report to *n* = 24. The injury mechanisms ranged from low brachial plexus injuries to the traumatic separation of the ulnar nerve at the elbow or wrist levels (the authors did not specify the type of brachial plexus injuries, whether they are avulsion, stretching or ruptures injuries, but all patients showed chronic impairment in C7-T1 territories), including leprosy sequelae in the ulnar nerve territory, crush, and burns. It also comprised large nerve defects after neuroma or neurofibroma resections, low ulnar nerve palsy, and sequelae of traumatic nerve separation following sharp traumatism or gunshot wound without any recovery after six months since the primary repair. The time before surgery varied from one to 252 months (the one month specific early neurotization was performed after a loss of nervous tissue at the elbow level in a 36-year-old patient, but the authors did not give any explanation about the early procedure timing). Although some of the surgeries were performed within the year following the injury, several authors described a median delay ranging from four months [13] to 67.6 months [14] (Table 2).

### 3.2. Surgical Procedures

**Figure 2:**
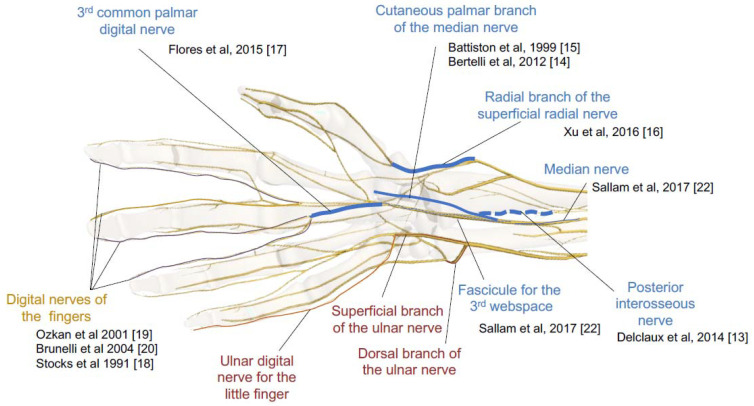
Summary of the ten surgical strategies. In blue: donor nerves. In red: recipient nerves at the wrist level. In yellow: recipient nerves at the hand level [13,14,15,16,17,18,19,20,22]. (Image created using ZygoteBody Professional— zygotebody.com).

In total, these nine studies described six donor nerves at the wrist level: the palmar branch of the median nerve, the cutaneous branch of the median nerve to the palm with or without fascicles of the ulnar digital nerve of the index finger (in case of size diameter mismatching) [15], the posterior interosseous nerve [13], the third common palmar digital nerve [17,21], the radial branch of the superficial radial nerve [16], the median nerve, and the fascicule for the third web space [22]. There are also three donor nerves at the hand level: the ulnar digital nerves of the index finger, and the radial or ulnar digital nerves of the long finger [18,19,20]. Three target sites were used: the sensory branch of the ulnar nerve (SBUN), the dorsal branch of the ulnar nerve (DoBUN), and the ulnar digital branch of the fifth digit (dVu) (Figure 2).

Regarding the suture type, preferences were for an end-to-end (ETE) coaptation of the nerves. However, some surgeons started to use an end-to-side (ETS) suture to restore the sensation of the SBUN territory [21]. Interestingly, Sallam et al. suggested coopting the distal stump of the donor fascicle to its main nerve trunk with an ETS suture in order to limit the donor zone deficit [22]. The characteristics of the studies included are summarized in Table 3.

### 3.3. Surgical Technique

**Figure 3:**
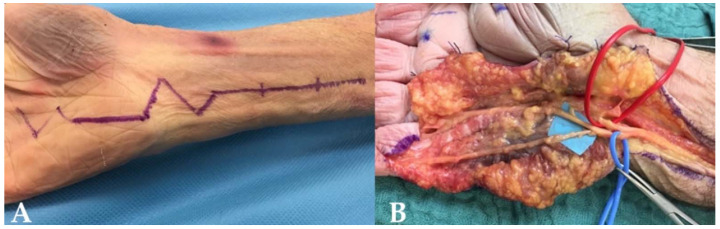
(**A**) The Taleisnik approach: along the axis of the ring finger, the incision reached the radial border of the flexor carpi ulnaris tendon, followed a zigzag pattern at the distal wrist crease. (**B**) Opening of the Guyon canal: The ulnar nerve was dissected, the SBUN was isolated in the blue loop, and the motor branch was separated (red loop). The incision exposed the terminal branches of the SBUN for the fourth and fifth digit.

We used Taleisnik’s approach [23,24] to expose the ulnar nerve at the wrist level (Figure 3A). The incision was made along the axis of the ring finger, over 10 cm above the pisiform bone, then reaching the radial border of the flexor carpi ulnaris (FCU) tendon. At the distal wrist crease, the incision followed a zigzag pattern, then ran into the palm following the palmar creases.

The ulnar neurovascular bundle was identified proximally in the distal forearm and released until it reached the Guyon canal. It was then retracted at the ulnar side and the hypothenar fascia of the Guyon canal was released, at the radial side of the pisiform bone. The SBUN and ulnar motor branches were then exposed and the SBUN was marked with a vessel loop. A small branch from the SBUN remained, innervating the palmaris brevis (Figure 3B).

The median nerve was identified in the distal forearm. The carpal tunnel had to be released so the subsequent transfers could be performed without tension.

#### 3.3.1. End-to-Side Reinnervation of the DoBUN, End-to-End Transfer of the Fascicule for the Third Web Space to the SBUN

**Figure 4:**
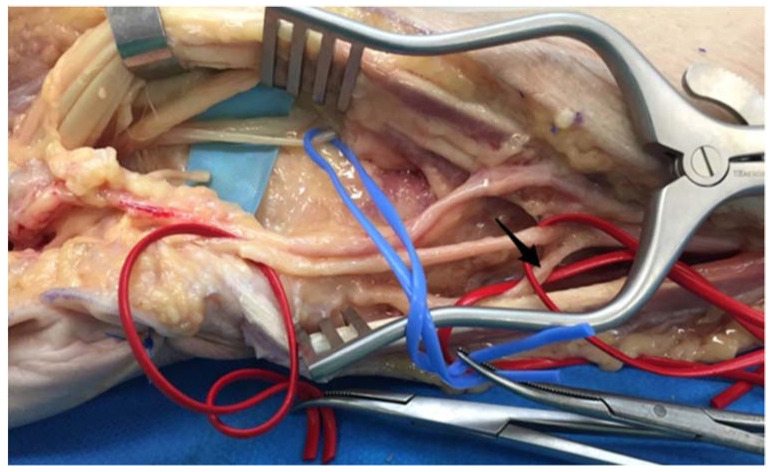
Isolation of the fascicle for the 3 web space and the DoBUN. The ulnar nerve was isolated in the red vessel loop. Between the flexor tendons, the median nerve was identified, and interfascicular dissection of the median nerve was carried out to isolate the fascicle for the third web space (blue loop).

The fascicle for the third web space was easily identified at the distal forearm thanks to the natural cleavage plane between this fascicle and the remaining median nerve. We had to make sure that this fascicule was the most ulnar one, avoiding the sensory fascicule for the first web space. After identification, we were able to stimulate it, in order to confirm the absence of motor function, and we then transected it. The DoBUN usually runs eight centimeters along the ulnar styloid. It has to be released until it crosses the FCU tendon (Figure 4). After proximal transection, the SBUN was sutured to the fascicule for the third web space. The DoBUN was then transected proximally and sutured to the remaining sensitive fibers of the median nerve in an end-to-side manner, after creating an epineural window at the coaptation level. This transfer was performed deep to the finger flexor tendons.

#### 3.3.2. End-to-End Transfer of the Palmar Cutaneous Branch of the Median Nerve to the SBUN

**Figure 5:**
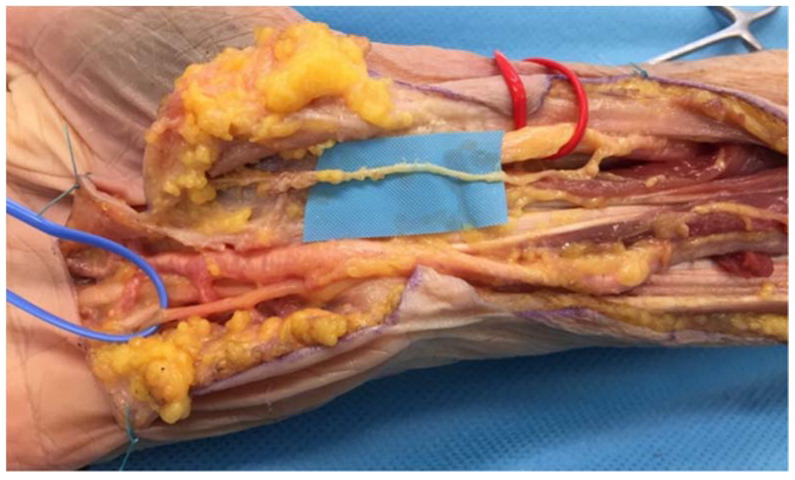
Release of the palmar cutaneous branch of the median nerve. The SBUN was isolated in the blue loop. The median nerve was identified (red loop) and the palmar cutaneous branch was released as far as possible.

The Taleisnik’s incision was extended over two centimeters along the pisiform bone, revealing the Guyon canal, which was opened, and the flexor retinaculum. The SBUN was transected proximally at its branching point. The cutaneous palmar branch of the median nerve lays on the flexor retinaculum, along the axis of the second web space (Figure 5). This branch was released until its distal part and was then transected and sutured to the distal stump of the SBUN. Bertelli et al. also suggested using the nerve after its division, harvesting the branch for the palm as a donor nerve, and suturing it to the digital nerve of the fifth digit [14].

#### 3.3.3. End-to-End Transfer of the Radial Branch of the Superficial Radial Nerve to the Palmar Sensory Fascicle of the Ulnar Nerve

**Figure 6:**
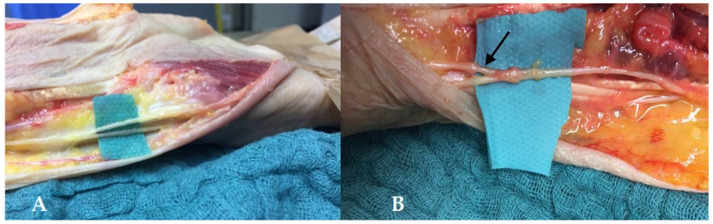
(**A**) The superficial radial nerve (SRN). The superficial radial nerve (SRN) gives two subdivisions. (**B**) The superficial branch of the radial nerve (SBRN). The most radial one gives a little thenar branch and the dorsal radial collateral nerve for the thumb (black arrow) or SBRN.

An incision was made on the radial side of the wrist and extended over seven centimeters proximally to the radial styloid. The superficial radial nerve (SRN) is located dorsolaterally to the brachioradialis (Figure 6A). We had to be careful to not confuse the latter with the superficial branch of the musculocutaneous nerve. The SRN was released as far as possible, following its division into the dorsal radial collateral nerve for the thumb (Figure 6B). This branch was then transected at the distal wrist crease. The fascia from the brachioradialis to the extensor carpi radialis was released to avoid potential shear stress after mobilization. Next, a Taleisnik’s incision was made on the ulnar side of the wrist. After decompression of the Guyon canal, the SBUN was identified and traced back proximally. At this point, Xu et al. suggest performing an interfascicular dissection of the ulnar nerve, until the SBUN fibers merged with the deep branch fibers (about five centimeters proximally to the distal wrist crease) [16]. The radial branch of the SRN was brought through a subcutaneous channel to the proximal stump of the SBUN, allowing us to perform an end-to-end transfer.

#### 3.3.4. Third Common Palmar Digital Nerve Transfer to the SBUN with an End-to-Side Suture

**Figure 7:**
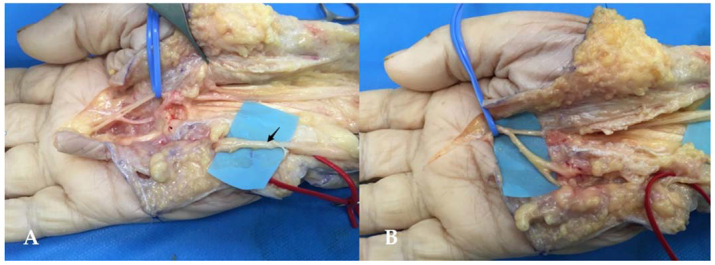
(**A**) Third common palmar digital branch of the median nerve. The third common palmar digital nerve was isolated in the blue loop. The SBUN is shown by the black arrow. (**B**) End-to-side (ETS) suture. The distal stump of the SBUN was transected in proximal and twisted 180° before the ETS suture with the third common palmar digital nerve.

We used the Taleisnik’s approach, extended into the palm following the creases in the third digital web space axis. First, the SBUN was identified at the level of Guyon’s canal and transected proximally. Then, the divisions of the median nerve were isolated at the level of the distal border of the carpal tunnel, which was opened (Figure 7A). The third common palmar digital nerve was isolated to perform an end-to-side (ETS) suture with the distal stump of the SBUN, which was twisted 180° (Figure 7B).

#### 3.3.5. Nerve Transfers on the Digital Branch of the Fifth Digit

**Figure 8:**
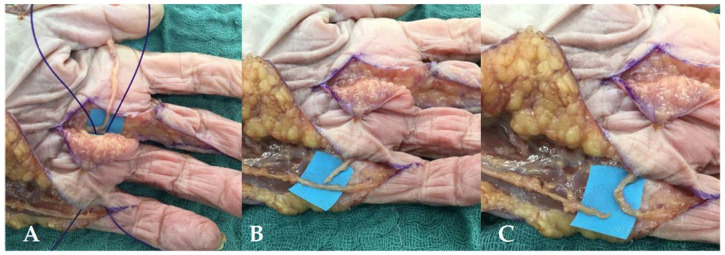
(**A**) Retrieving the ulnar branch of the third median digital nerve. We used a PDS loop to retrieve the donor nerve through a subcutaneous channel. (**B**) Isolation of the donor nerve. We exposed the level of transection of the digital nerve of the small finger. (**C**) End-to-end (ETE) suture on the digital branch of the small finger. The digital branch of the small finger was transected proximally and an ETE suture was performed at the distal crease level of the palm.

A hemi-Brunner’s approach was used to release the donor and receiver nerves. The donor was then transected at the level of the distal interphalangeal joint. Through a subcutaneous channel, we transferred it to the digital nerve of the small finger at the distal crease level of the palm with an end-to-end (ETE) suture (Figure 8A,C).

#### 3.3.6. Neurotization of the SBUN by the Posterior Interosseous Nerve (PIN)

**Figure 9:**
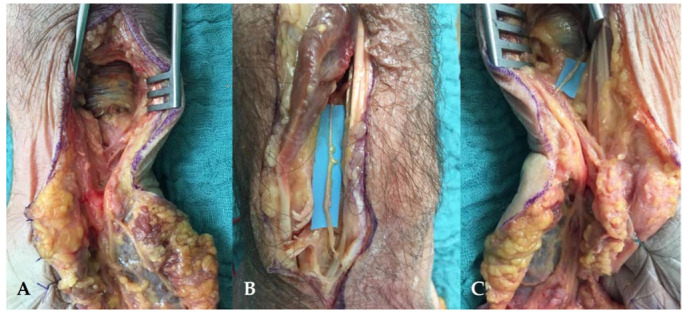
(**A**) Exposure of the pronator quadratus. The pronator quadratus was identified and an incision was made to expose the interosseous membrane. (**B**) Dorsal approach of the DRUJ. The PIN was released over 5.5 cm and transected distally. (**C**) Retrieving the PIN at the palmar side of the forearm. The PIN was passed through the interosseous membrane in order to make the ETE suture with the sensory fascicle of the SBUN.

The ulnar nerve was identified at the level of Guyon’s canal, after a Taleisnik’s approach as described above. Using a distal to proximal dissection, the SBUN was released until its sensitive nerve fibers merged with the motor fibers of the deep branch of the ulnar nerve, about five centimeters proximal to the distal wrist crease. Retracting the neurovascular bundle at the ulnar side, the ulnar border of the pronator quadratus muscle was exposed (Figure 9A). An incision was made along the muscle to prepare a tunnel through the interosseous membrane.

A dorsal incision was used, centered on the distal radio-ulnar joint. The posterior interosseous nerve (PIN) runs on the ulnar side of the Lister’s Tubercle, between the extensor pollicis longus tendon and the fourth compartment of the extensor tendons. It was released over 5.5 cm at least and transected as distally as possible (Figure 9B). The PIN was then retrieved through the interosseous membrane (Figure 9C). Afterward, we sutured the PIN to the sensory fascicle of the SBUN in an end-to-end suture.

### 3.4. Functional Results

#### 3.4.1. BMRC Assessment

The follow-up period varied between 15 and 119 months, with a majority of good or even complete recoveries in the receiving territory, scaled S3+ or S4, for every technique, except in the Brunelli et al. study [20]. The functional outcomes from the studies included are summarized in Table 4.

#### 3.4.2. Donor Site Morbidity

Regarding the donor site deficit, most authors did not report any significant impairment, such as paresthesia or dysesthesia, nor excessive discomfort. Xu et al., noted a small zone of paresthesia at the radial dorsal side of the thumb, which disappeared within 12 months [16]. Similarly, after a digital nerve transfer, many patients regained sensation to some extent [19]. In the series by Sallam et al., most patients have regained deep pain sensibility in the donor zone [22]. No neuroma has been noticed. The donor site impairments are summarized in Table 5.

## 4. Discussion

### 4.1. Fascicular Topography

Regarding the intraneural dissection, the surgeon must be familiar with the different nerve topographies, as this makes the procedure more reliable. Moore et al. described ulnar fascicles as not being easily or consistently separated at the wrist, making the outcomes more unpredictable. Some authors report that visual neurolysis of these fascicules could be helped by the presence of noticeable microvessels within the epineurium, corresponding to the demarcation between motor and sensory fascicles [24,25].

Median nerve topography has been studied by Planitzer et al. At the middle—distal third junction of the forearm, the fascicle for the third web space is easily separated from the remainder of the nerve [26]. Twenty-one distal median nerves from ethanol-glycerin-fixed body donors were investigated. After removing the epineurium they were assigned to four quadrants. Fascicles supplying the palmar ulnar side of the third digit and palmar radial side of the fourth digit originated from the nerves’ ulno-palmar part in 63.2 and 65% of cases, respectively. Moreover, in this study, fascicles of the third web space were located exclusively in the ulnar part of the median nerve, and only 5% of the fascicles supplying the fourth digit came from the radial part [26]. This regularity makes the outcomes more reliable if the harvested fascicle always comes from the ulnar side of the median nerve.

### 4.2. Suture: Diameter Mismatch and Shear Stress

In our experience, following cadaveric studies, there was a mismatch regarding nerve diameter in two cases: end-to-end transfers of the PIN and the radial branch of the SBRN onto the palmar sensory fascicle for the fourth and fifth finger. These two donor nerves have a smaller diameter than the target one. This mismatch was not reported by Delclaux (PIN transfer) [13] or Xu (SBRN transfer) [16]. In an anatomical study, Schenck et al. suggested harvesting the SBRN proximally to its first bifurcation [27]. Then, starting distally, the SBUN and DoBUN were separated carefully over a length of 49.4 ± 5.5 mm to avoid the mismatching of the motor and sensory axons. Their strategy differs from that of Xu et al. on two points [16]: the level of SBRN harvesting (distally or proximally to the SBRN bifurcation, which is found about three centimeters proximally to the styloid process of the radius), and the transposition of the SBRN [27]. Xu et al. used a subcutaneous channel [16], which in our opinion could damage the nerve, regarding the shear stress when SBRN emerges from the aponeurosis between the brachioradialis muscle and extensor carpi radialis longus muscle, approximately seven to 11 cm proximally to the radial styloid process. Schenck et al. passed the harvested SBRN under the brachioradialis, flexor carpi radialis, and flexor pollicis longus muscles, which helped to prevent shear stress. According to them, the axon ratio was 1:1.4 for the SBRN to SBUN transfer using this technique, and the axon density of the SBRN exceeded that of the SBUN [27]. However, harvesting the SBRN proximal to its bifurcation [27] leads to a more significant donor site defect than using only the radial branch of the SBRN. In addition, mobilizing the PIN through the interosseous membrane also puts it at risk of shear stress, but the length of the nerve harvested did not allow us to choose another transposition.

**Figure 10:**
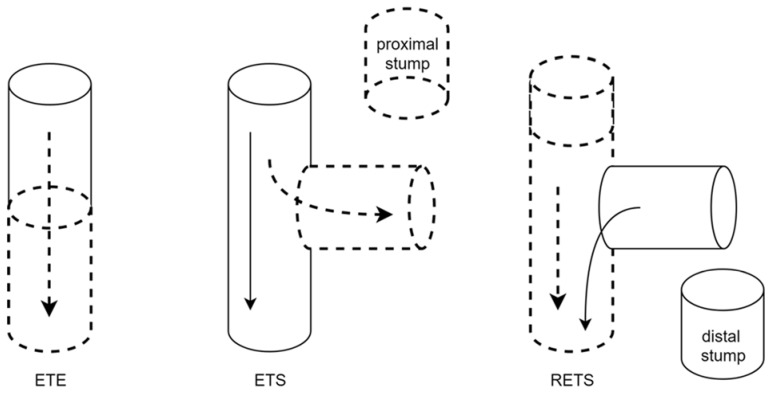
Illustration of the types of nerve transfer (adapted from Lee et Wolfe 2012 [31]). Full line: donor nerve. Dash line: receiving nerve. ETE: end-to-end suture. ETS: end-to-side suture. The axons sprout from the side of the donor nerve into the end of the recipient nerve. RETS: reverse end-to-side suture. The donor axons enter the receiving nerve from its side in order to enhance the axonal sprouting process of the repaired receiving nerve.

Three different methods for nerve coaptation have been described (Figure 10). Among them, the most straightforward one is the end-to-end nerve coaptation (ETE). However, due to complete transection, the morbidity in the donor territory becomes the major drawback. Thus, some authors would rather do an end-to-side suture (ETS), with the distal end of the recipient nerve coopted onto the donor nerve after a partial epineurotomy [28]. Interestingly, no significant long-term changes in functional, electrophysiological, or morphological properties of the donor nerve have been described after ETS nerve coaptation in rats [29]. More recently, some experimental studies focused on reverse-end-to-side suturing (RETS), which aims to increase axonal sprouting in nerve injuries when functional recovery is predicted [30]. In this technique, after a microsurgical epineural repair, the proximal stump of the injured nerve is free for potential regeneration. The donor nerve is coopted to the side of the distal targeted nerve through an epineural window.

### 4.3. ETS: With or without an Epineural Window

In this review, two authors used the ETS suture: Flores et al. performed it without any epineural window [21], contrary to Sallam et al. [22]. According to an experimental work on rats, axotomy or compression are required for axonal sprouting following an ETS neurorrhaphy [28]. However, Flores et al., suggested that because of the very thin connective tissue of the digital nerves, neurolysis combined with epineurium injuries caused by the sutures themselves may be enough for the axonal sprouting process to take place [21]. This strategy is coherent with some animal study outcomes, demonstrating that collateral sprouting could occur from intact axons with an ETS suture [32]. Interestingly, the ETS suture could be used to prevent donor zone deficit [28,29,30]. In this way, Moore suggested that the distal stump of the fascicle of the third web space could be sutured to the median nerve in an ETS fashion, combined with the proximal stump transfer to the SBUN [33].

### 4.4. Sensory Nerve Regeneration

Surprisingly, contrary to the axiom “time is muscle”, sensory transfers appear to be successful even years after the injury [19]. There are several types of mechanoreceptors, and their behavior varies following denervation. All of them undergo slow, progressive degeneration but the time course of this process remains unclear. Cutaneous sensory nerve formations such as Merkel cells can undergo quick and complete degeneration, whereas Meissner corpuscles are subjected to slow and partial degradation after denervation [34]. Nevertheless, some animal studies have shown that about 40% of Merkel cells survived after denervation. Moreover, reinnervation may induce differentiation of new Merkel cells in places where their numbers have become reduced after denervation [35]. Furthermore, auxiliary structures such as terminal Schwann cells play a key role in sensory nerve regeneration: the continuous basal laminae between terminal Schwann cells and the myelin-forming Schwann cells in the nerve fibers is known to form a pathway for regrowing axons [36]. In addition, their trophic independence from the sensory terminals, which rapidly disappears through Wallerian degeneration after a severed nerve, allows them to survive after denervation [37]. Coulet et al. suggested that sensory recovery reaches a plateau between three and four years after a lower lesion, and four to five years in the case of an upper lesion [34].

Regeneration was also studied at a more central level. For dorsal root ganglion primary sensory neurons, the peripheral branch which innervates the sensory organs regenerates spontaneously after injury. Conversely, the central branch, which enters the spinal cord and terminates in the brain, does not, because of myelin-associated inhibitory molecules in their environment. However, thanks to a phenomenon known as conditioning peripheral lesion, a peripheral branch injury can activate the intrinsic growth capacity and overcome the myelin-associated inhibitory effect [38].

### 4.5. Sensory Assessment

Many sensory assessment tools have been described, mainly focused on the perception of the cutaneous threshold (Semmes-Weinstein test), whereas protective sensation relies more on tactile gnosis (two-point discrimination (2PD) test, either static -S2PD- or moving -M2PD-). Other functional sensory tests evaluate the shape, the texture identification, the vibration and temperature discriminations [10]. Among the sensory assessment scales, the British Medical Research Council Scale modified by Mackinnon and Dellon is the most commonly used [7]. Not only tactile gnosis (based on 2PD) is evaluated, but it also assesses deep and superficial pain recoveries, along with tactile sensibility and abnormal over-response. However, 2PD outcomes in nerve repair studies were reported to be variable, as neither the pressures applied, nor the testing protocol are standardized. In most of the selected studies, data are lacking regarding the chosen 2PD protocol: the descending-ascending width of the caliper, or the descending width with randomization [39], the device: Diskriminator [40] or paperclip with bending tips at 90°, and the penetration depth related to the pressure applied [41,42], which can affect the test’s reproducibility. Moreover, in order to obtain a more complete evaluation, qualitative data including pain and the subjects’ assessments of improvement in function should be associated [43].

### 4.6. Neurotization vs. Nerve Grafting

Of these studies, two authors focused on neurotization functional outcomes versus sural nerve grafting. Flores et al., compared the reinnervation of the SBUN using the third common palmar digital nerve in ETS (*n* = 15) versus sural nerve grafting (*n* = 20). There was no significant difference: 30% of the nerve grafting patients scored S3+/S4, and 40% of the nerve transfer group (*p* = 0.071) [17] scored the same. Sallam et al., compared reinnervation of the SBUN with the third web space sensory fascicle of the median nerve (ETS) associated with an ETS suture of the DoBUN to the median nerve (*n* = 24) versus sural nerve grafting (*n* = 28). At the final follow-up, the two groups had regained the same sensory function: 53.6% of the nerve grafted patients scored S3 or more, versus 58.3% in the nerve transfer group [22].

### 4.7. Reeducation & Cortical Adaptation

Flores and Sallam both noted that one of the drawbacks of nerve transfers comes from sensory crossed innervation [17,22], which requires cortical adaptation [44]. Rehabilitation is enhanced by sensory reeducation programs, as proposed by Dellon & Jabaley [45]. Pain and temperature perceptions are the first stimuli to be regained, followed by the perception of slow vibrations and moving touch stimuli, and finally quick vibrations and tactile gnosis. According to these authors, respecting the sensory recovery timetable is necessary for avoiding frustration and failure by initiating an exercise before the recovery of the appropriate fiber-receptor. They suggested beginning the early phase reeducation four to six months after an ulnar nerve suture at the level of the wrist when the recovery reaches the proximal phalanx. The late phase reeducation focuses on tactile gnosis and should begin six to eight months after the ulnar nerve suture at the wrist level. However, some exciting recent works focused on early rehabilitation starting in the first week after surgery in order to improve outcomes after nerve repair. Thanks to guided plasticity training through mirror visual feedback and observation touch, patients showed better discriminative touch six months after surgery than the control group, who only started the rehabilitation when the outgrowing axons had reinnervated the skin at the fingertip level [46].

### 4.8. Limitations of the Study

#### 4.8.1. Study Population

As these surgical procedures are quite rare, the population study appears very diverse, ranging from low brachial plexus injuries, to traumatic severed nerves or leprosy sequelae. Our inclusion criteria thus combine different situations, but all of them share the following common feature: these nerve damages cannot be repaired in situ. Either the extensive crush or avulsion primary injury lead to a large nerve defect after debridement of the viable margins, or the nerve damage is very proximal with a long distance from the targeted sensory organ, increasing the risk of misrouting during the excessively long nerve regrowth.

Regarding leprosy nerve damage, the irreversible injury in the internal axonal structure impairs the identification of viable margins. Pathophysiology of nerve damage in leprosy relies on *M. leprae* molecular affinity to Schwann cells, with early nerve demyelination mediated by ErB2 receptor tyrosine kinase signaling. Another in vitro study suggested that *M. Leprae* triggers glial cell proliferation and inflammatory response [47] leading to the appearance of immune-mediated lesions [48]. Keeping in mind that this condition is very distinct from the demyelination and progressive axonal degradation occurring during the Wallerian degeneration process after a complete transection of a peripheral nerve [38], very few nerve transfer procedures have been described in leprosy. Yet, while most of the reconstructions after Hansen disease motor sequelae rely on tendon transfers, sensory restoration is still based on nerve transfers [49]. Nerve damage sequelae in leprosy could be considered as a relative indication for neurotization. However, as the outcomes in this particular condition range from S3 to S3+ after digital nerve transfer (i.e., dIIIu to dVu), this could provide a palliative option [19].

Furthermore, regarding brachial plexus injuries, data are sometimes lacking concerning additional surgical procedures other than sensory neurotization [14,18,20]. Thus, care should be taken when interpreting global hand functional results. Only Xu et al. described a distal nerve transfer in order to restore finger flexion and extension. The brachialis motor branch has been transferred to the finger flexor fascicles of the median nerve and the supinator motor branch to the posterior interosseous nerve. However, despite a partial sensory recovery on the ulnar aspect of the forearm, patients still lack protective sensibility at the ulnar side of the hand after a 33.3 months interval from this surgery, which led the authors to propose to them the distal sensory neurotization of their ulnar nerve [16].

Overall, due to cases heterogeneity and sample fluctuation regarding the few number of patients in most of the studies, care should be taken while generalizing their results. Nonetheless, the Salam et al. (*n* = 24) [17] and Flores et al. (*n* = 15) [18] studies account for 48% of all patients, and their results might be more reliable. This review underlined the heterogeneity of this study population. In our opinion, it could be representative for a widespread neurotization application in all traumatic nerve injury patients with a large nerve defect, as long as the damage cannot be repaired in situ without tension, with poor margins unsuitable for a nerve graft, especially if it is located more than 60 cm from the tip of the finger [50].

#### 4.8.2. Surgical Strategy and Donor Site Impairment

Some can argue that the functional and ethical principle of using radial-sided nerves for potentially restoring the sensory function of the ulnar side of the hand can be criticized. Indeed, it is commonly admitted that the thumb, the radial aspect of the index finger, and the ulnar border of the small finger are the most important areas among relative values of sensibility in the hand. Yet, as to Brunelli’s choice, reporting a dIIIr transfer to the dVu [20], this procedure could impair the pinch sensibility between the thumb and the radial side of the middle phalanx of the third digit. Moreover, in lower type brachial plexus injuries, sensation in the DoBUN territory is impaired. Hence, while harvesting part of the SRN, care was taken to only use its radial branch, which gives the dorso-proximal thumb sensibility. As Xu et al. did preserve the ulnar branch of the SRN, they prevent the complete anesthesia on the dorsal side of the hand [16].

Moreover, in most of the studies, the donor site morbidity has been assessed only by subjective questionnaires regarding pain, dysesthesia, or impairment in daily life activities. Further investigations could use the Semmes-Weinstein monofilament test to evaluate the loss of protective sensation at the donor site [10]. In order to prevent this morbidity, Sallam et al. described an end-to-side repair from the distally divided end of the third web space fascicle to the intact median nerve. They reported that sensory recovery of S1 in the third web space was achieved in 22 of 24 patients and S2 in 2 of 24. However, they did not specify the time of assessment after the surgery, nor the testing location [22].

Finally, compensatory circuits generated by collateral sprouting might play a role in the rather good subjective outcome of the patients. Given that collateral sprouting is triggered by signals from damaged fibers during the Wallerian degeneration process, it should definitely be considered when the nerve transfer has been done early after the initial injury [51]. However, when the recipient nerve has been reinnervated after chronic impairment, this phenomenon is less likely to occur at the targeted site.

## 5. Conclusions

Overall, considering the data available on sensory neurotization of the ulnar nerve, these techniques seem to be interesting palliative options. Knowing that nerve sprouting is about 0.5 to one millimeter per day, neurotization at the level of the wrist or the hand may decrease the reinnervation time, which may be useful for patients who cannot withstand further surgery because of the excessively long restoration time. There are multiple donor nerves, allowing surgeons to adapt their strategy for each complex case after ulnar lesion at, or proximal to, the elbow. Even if using these techniques remains rare, their outcomes suggest that anesthesia sequelae in the ulnar territory could be improved.

## Figures and Tables

**Table 1:** Classification of sensory recovery according to the Nerve injuries committee of the British Medical Research Council (BMRC), modified by Mackinnon and Dellon (adapted from Wang et al., 2013 [10]).

BMRC Scale of Sensory Recovery Modified by Mackinnon and Dellon	
S0	Anesthesia
S1	Deep pain sensibility
S1+	Superficial pain sensibility
S2	Pain and some touch sensibility
S2+	Pain and some touch sensibility with some over response
S3	Pain and some touch sensibility without over response—S2PD > 15 mm, M2PD > 7 mm
S3+	Sensory localization—S2PD between 7 to 15 mm, M2PD between 4 to 7 mm
S4	Complete recovery—S2PD < 6 mm, M2PD < 3 mm

**Table 2:** Characteristics of the studies included assessing sensory neurotization of the ulnar nerve.

Study/*n*	Etiology	Median Delay Before Surgery
Delclaux et al., 2014 [13], *n* = 1	Traumatic separation at the elbow level	12 M
Battiston & Lanzetta et al., 1999 [15], *n* = 7	Traumatic separation above or at the elbow level	4 M (1–6)
Xu et al., 2016 [16], *n* = 4	Low brachial plexus injury	20.5 M (16–78)
Flores et al., 2015 [17], *n* = 15	Traumatic separation at the elbow level	7.1 M * (1–8)
Sallam et al., 2017 [18], *n* = 24	Section at the elbow level	9.4 M * (6–18)
Ozkan et al., 2001 [19], *n* = 10	Leprosy, crush, burn	60 M (3–252)
Stocks et al., 1991 [18], *n* = 9	Distal ulnar nerve injury	12 M (7–120)
Brunelli et al., 2004 [20], *n* = 2	Brachial plexus injury	NA
Bertelli et al., 2012 [14], *n* = 8	Low brachial plexus injury	4 M (3–28)

M: Month, Y: Year. NA: non-available, *: mean value.

**Table 3:** Surgical procedures.

Study/*n*	Donor Nerve	Recipient Nerve	Suture Type
Delclaux et al., 2014 [13], *n* = 1	PIN	SBUN (fascicle)	ETE
Battiston & Lanzetta et al., 1999 [15], *n* = 7	Cutaneous branch of the median nerve	SBUN	ETE
Xu et al., 2016 [16], *n* = 4	Radial branch of the SBRN	SBUN (fascicle)	ETE
Flores et al., 2015 [17], *n* = 15	Third palmar common digital nerve	SBUN	ETS
Sallam et al., 2017 [22], *n* = 24	Fascicle for the third web space	SBUN	ETE
	Median nerve	DoBUN	ETS
	Median nerve	Fascicule for the third web space	ETS
Ozkan et al., 2001 [19], *n* = 10	dIIIu	dVu	ETE
	dIIu		
Stocks et al., 1991 [18], *n* = 9	dIIIu	dVu	ETE
Brunelli et al., 2004 [20], *n* = 2	dIIr	dVu	ETE
Bertelli et al., 2012 [14], *n* = 8	Palmar cutaneous branch of the median nerve	dVu	ETE
	Cutaneous branch of the median nerve to the palm		

ETE: end-to-end suture. ETS: end-to-side suture. dIIu: ulnar digital nerve of the index finger, dIIIr: radial digital nerve of the long finger, dIIIu: ulnar digital branch of the long finger, dVu: ulnar digital nerve of the small finger. PIN: posterior interosseous nerve. SBRN: superficial branch of the radial nerve. SBUN: sensory branch of the ulnar nerve. DoBUN: dorsal branch of the ulnar nerve.

**Table 4:** BMRC assessment of sensory recovery.

Study/*n*	Sensory Recovery		Median Follow-Up
Delclaux et al., 2014 [13], *n* = 1	S2 at the base of the 5th finger		18 M
Battiston & Lanzetta et al., 1999 [15], *n* = 7	1/7≤ S3 5/7 S3+ 1/7 S4		18 M (12–24)
Xu et al., 2016 [16], *n* = 4	3/4 S3 1/4 S3+		18.5 M (18–27)
Flores et al., 2015 [17], *n* = 15	9/15 ≤ S3 6/15 S3+/S4		24.3 M * (15–38)
Sallam et al., 2017 [22], *n* = 24	3/24 S1 7/24 S2 8/24 S3 2/24 S3+ 4/24 S4		28.6 M* (24–38)
Ozkan et al., 2001 [19], *n* = 10	dIIIu	1/5 S3 3/5 S3+ 1/5 S4	75 M (58–119)
	dIIu	3/5 S3+ 2/5 S4	76.5 M (58–94)
Stocks et al., 1991 [18], *n* = 9	2/9 ≤ S3 4/9 S3+ 3/9 S4		48.5 M (15–96)
Brunelli et al., 2004 [20], *n* = 2	S1 S2+		NA
Bertelli et al., 2012 [14], *n* = 8	Palmar cutaneous branch of the median nerve	3/8 S3	36 M (24–36)
	Cutaneous branch of the median nerve to the palm	2/8 S3 3/8 S3+	27 M (24–48)

The BMRC scale for sensation is categorized from S0 to S4: S0 refers to the absence of sensitivity sensation, while S4 is complete recovery with S2PD between 2–6 mm and M2PD: 2–3 mm. The detailed scale is developed in Table 1 [10]. M: months. *: mean value.

**Table 5:** Donor site morbidity.

Study/*n*	Donor Site	Complication
Delclaux et al., 2014 [13], *n* = 1	PIN	No negative sensory consequence
Battiston & Lanzetta et al., 1999 [15], *n* = 7	Cutaneous branch of the median nerve	No excessive discomfort from the anesthetic area: 3 cm^2^ in the thenar region
Xu et al., 2016 [16], *n* = 4	Radial branch of the SBRN	Small zone of paresthesia which disappeared within 12 months (radial dorsal side of the thumb)
Flores et al., 2015 [17], *n* = 15	Third palmar common digital nerve	NA
Sallam et al., 2017 [22], *n* = 24	Fascicle for the third web space	22/24: S1, 2/24: S2
	Median nerve	
	Median nerve	
Ozkan et al., 2001 [19], *n* = 10	dIIIu	No paresthesia or dysesthesia at the donor site
	dIIu	
Stocks et al., 1991 [18], *n* = 9	dIIIu	No significant impairment
Brunelli et al., 2004 [20], *n* = 2	dIIr	NA
Bertelli et al., 2012 [14], *n* = 8	Palmar cutaneous branch of the median nerve	No donor site deficit
	Cutaneous branch of the median nerve to the palm	

NA: not available.

## References

[1] Lad S.P., Nathan J.K., Schubert R.D., Boakye M. (2010). Trends in Median, Ulnar, Radial, and Brachioplexus Nerve Injuries in the United States. Neurosurgery.

[2] Omer G.E. (1982). Reconstructive Procedures for Extremities with Peripheral Nerve Defects. Clin. Orthop. Relat. Res..

[3] Vordemvenne T., Langer M., Ochman S., Raschke M., Schult M. (2007). Long-Term Results after Primary Microsurgical Repair of Ulnar and Median Nerve Injuries. Clin. Neurol. Neurosurg..

[4] Millesi H., Meissl G., Berger A. (1976). Further Experience with Interfascicular Grafting of the Median, Ulnar, and Radial Nerves. J. Bone Jt. Surg..

[5] Bertelli J.A., Ghizoni M.F., Loure Iro Chaves D.P. (2011). Sensory Disturbances and Pain Complaints after Brachial Plexus Root Injury: A Prospective Study Involving 150 Adult Patients. Microsurgery.

[6] Ruchelsman D.E., Price A.E., Valencia H., Ramos L.E., Grossman J.A.I. (2010). Sensory Restoration by Lateral Antebrachial Cutaneous to Ulnar Nerve Transfer in Children with Global Brachial Plexus Injuries. Hand.

[7] Novak C.B., Kelly L., Mackinnon S.E. (1992). Sensory Recovery after Median Nerve Grafting. J. Hand Surg..

[8] Dellon A.L. (1978). The Moving Two-Point Discrimination Test: Clinical Evaluation of the Quickly Adapting Fiber/Receptor System. J. Hand Surg..

[9] Jerosch-Herold C. (1993). Measuring Outcome in Median Nerve Injuries. J. Hand Surg..

[10] Wang Y., Sunitha M., Chung K.C. (2013). How to Measure Outcomes of Peripheral Nerve Surgery. Hand Clin..

[11] Menier C., Forget R., Lambert J. (1996). Evaluation of two-point discrimination in children: Reliability, effects of passive displacement and voluntary movements. Dev. Med. Child Neurol..

[12] Davidge K.M., Mackinnon S.E. (2013). The Supercharge End-to-Side Anterior Interosseous to Ulnar Motor Nerve Transfer for Restoring Intrinsic Function: Clinical Experience. J. Hand Surg..

[13] Delclaux S., Aprédoaei C., Mansat P., Rongières M., Bonnevialle P. (2014). Case Report: Double Nerve Transfer of the Anterior and Posterior Interosseous Nerves to Treat a High Ulnar Nerve Defect at the Elbow. Chir. De La Main.

[14] Bertelli J.A. (2012). Distal Sensory Nerve Transfers in Lower-Type Injuries of the Brachial Plexus. J. Hand Surg..

[15] Battiston B., Lanzetta M. (1999). Reconstruction of High Ulnar Nerve Lesions by Distal Double Median to Ulnar Nerve Transfer. J. Hand Surg..

[16] Xu B., Dong Z., Zhang C.-G., Gu Y.-D. (2016). Transfer of the Radial Branch of the Superficial Radial Nerve to the Sensory Branch of the Ulnar Nerve for Sensory Restoration after C7-T1 Brachial Plexus Injury. J. Plast. Reconstr. Aesthetic Surg..

[17] Flores L. (2015). Comparative Study of Nerve Grafting versus Distal Nerve Transfer for Treatment of Proximal Injuries of the Ulnar Nerve. J. Reconstr. Microsurg..

[18] Stocks G.W., Cobb T., Lewis R.C. (1991). Transfer of Sensibility in the Hand: A New Method to Restore Sensibility in Ulnar Nerve Palsy with Use of Microsurgical Digital Nerve Translocation. J. Hand Surg..

[19] Özkan T., Özer K., Gülgönen A. (2001). Restoration of Sensibility in Irreparable Ulnar and Median Nerve Lesions with Use of Sensory Nerve Transfer: Long-Term Follow-up of 20 Cases. J. Hand Surg..

[20] Brunelli G.A. (2004). Sensory Nerves Transfers. J. Hand Surg..

[21] Flores L.P. (2011). Distal Anterior Interosseous Nerve Transfer to the Deep Ulnar Nerve and End-to-Side Suture of the Superficial Ulnar Nerve to the Third Common Palmar Digital Nerve for Treatment of High Ulnar Nerve Injuries. Arq. Neuropsiquiatr..

[22] Sallam A.A., El-Deeb M.S., Imam M.A. (2017). Nerve Transfer Versus Nerve Graft for Reconstruction of High Ulnar Nerve Injuries. J. Hand Surg..

[23] Taleisnik J. (1973). The Palmar Cutaneous Branch of the Median Nerve and the Approach to the Carpal Tunnel. JBJS.

[24] Brown J.M., Yee A., Mackinnon S.E. (2009). Distal Median to Ulnar Nerve Transfers to Restore Ulnar Motor and Sensory Function within the Hand. Neurosurgery.

[25] Patterson J.M.M. (2016). High Ulnar Nerve Injuries. Hand Clin..

[26] Planitzer U., Steinke H., Meixensberger J., Bechmann I., Hammer N., Winkler D. (2014). Median Nerve Fascicular Anatomy as a Basis for Distal Neural Prostheses. Ann. Anat. Anat. Anz..

[27] Schenck T.L., Lin S., Stewart J.K., Koban K.C., Aichler M., Rezaeian F., Giunta R.E. (2016). Sensory Reanimation of the Hand by Transfer of the Superficial Branch of the Radial Nerve to the Median and Ulnar Nerve. Brain Behav..

[28] Hayashi A., Pannucci C., Moradzadeh A., Kawamura D., Magill C., Hunter D.A., Tong A.Y., Parsadanian A., Mackinnon S.E., Myckatyn T.M. (2008). Axotomy or Compression Is Required for Axonal Sprouting Following End-to-Side Neurorrhaphy. Exp. Neurol..

[29] Kovačič U., Tomšič M., Sketelj J., Bajrović F.F. (2007). Collateral Sprouting of Sensory Axons after End-to-Side Nerve Coaptation—A Longitudinal Study in the Rat. Exp. Neurol..

[30] Kale S.S., Glaus S.W., Yee A., Nicoson M.C., Hunter D.A., Mackinnon S.E., Johnson P.J. (2011). Reverse End-to-Side Nerve Transfer: From Animal Model to Clinical Use. J. Hand Surg..

[31] Lee S.K., Wolfe S.W. (2012). Nerve Transfers for the Upper Extremity: New Horizons in Nerve Reconstruction. J. Am. Acad. Orthop. Surg..

[32] Lundborg G., Zhao Q., Kanje M., Danielsen N., Kerns J.M. (1994). Can Sensory and Motor Collateral Sprouting Be Induced from Intact Peripheral Nerve by End-to-Side Anastomosis?. J. Hand Surg..

[33] Moore A.M., Franco M., Tung T.H. (2014). Motor and Sensory Nerve Transfers in the Forearm and Hand. Plast. Reconstr. Surg..

[34] Coulet B., Chammas M., Daussin P.-A., Lazerges C., Lacombe F., César M., Domergue S., Bacou F., Micallef J.-P. (2007). Dégénérescence et régénération des nerfs périphériques et des effecteurs musculaires et sensitifs. Lésions Traumatiques Des Nerfs Périphériques.

[35] Nurse C.A., Macintyre L., Diamond J. (1984). Reinnervation of the Rat Touch Dome Restores the Merkel Cell Population Reduced after Denervation. Neuroscience.

[36] Idé C. (1977). Development of Meissner Corpuscle of Mouse Toe Pad: Mouse meissner corpuscle development. Anat. Rec..

[37] Dubový P., Aldskogius H. (1996). Degeneration and Regeneration of Cutaneous Sensory Nerve Formations. Microsc. Res. Tech..

[38] Chen Z.-L., Yu W.-M., Strickland S. (2007). Peripheral Regeneration. Annu. Rev. Neurosci..

[39] Zimney K., Dendinger G., Engel M., Mitzel J. (2022). Comparison of Reliability and Efficiency of Two Modified Two-Point Discrimination Tests and Two-Point Estimation Tactile Acuity Test. Physiother. Theory Pract..

[40] Mackinnon S.E., Dellon A.L. (1985). Two-Point Discrimination Tester. J. Hand Surg..

[41] Lundborg G., Rosén B. (2004). The Two-Point Discrimination Test—Time For a Re-Appraisal?. J. Hand Surg..

[42] Yokota H., Otsuru N., Kikuchi R., Suzuki R., Kojima S., Saito K., Miyaguchi S., Inukai Y., Onishi H. (2020). Establishment of Optimal Two-Point Discrimination Test Method and Consideration of Reproducibility. Neurosci. Lett..

[43] Rosén B., Lundborg G. (2000). A Model Instrument for the Documentation of Outcome after Nerve Repair. J. Hand Surg..

[44] Chemnitz A., Andersson G., Rosén B., Dahlin L.B., Björkman A. (2013). Poor Electroneurography but Excellent Hand Function 31 Years after Nerve Repair in Childhood. Neuro. Rep..

[45] Dellon A.L., Jabaley M.E. (1982). Reeducation of Sensation in the Hand Following Nerve Suture. Clin. Orthop. Relat. Res..

[46] Rosén B., Vikström P., Turner S., McGrouther D.A., Selles R.W., Schreuders T.A.R., Björkman A. (2015). Enhanced Early Sensory Outcome after Nerve Repair as a Result of Immediate Post-Operative Re-Learning: A Randomized Controlled Trial. J. Hand Surg. Eur. Vol..

[47] Wilder-Smith E.P., Van Brakel W.H. (2008). Nerve Damage in Leprosy and Its Management. Nat. Rev. Neurol..

[48] Aarão T.L.D.S., de Sousa J.R., Falcão A.S.C., Falcão L.F.M., Quaresma J.A.S. (2018). Nerve Growth Factor and Pathogenesis of Leprosy: Review and Update. Front. Immunol..

[49] Agarwal P., Shukla P., Sharma D. (2018). Saphenous Nerve Transfer: A New Approach to Restore Sensation of the Sole. J. Plast. Reconstr. Aesthetic Surg..

[50] Vastamkki M., Kallio P.K., Solonen K.A. (1993). The Results of Secondary Micros Ulnar Nerve Injury. J. Hand Surg..

[51] Lemaitre D., Hurtado M.L., De Gregorio C., Oñate M., Martínez G., Catenaccio A., Wishart T.M., Court F.A. (2020). Collateral Sprouting of Peripheral Sensory Neurons Exhibits a Unique Transcriptomic Profile. Mol. Neurobiol..

